# Comparing the Rates of Early Childhood Victimization across Sexual Orientations: Heterosexual, Lesbian, Gay, Bisexual, and Mostly Heterosexual

**DOI:** 10.1371/journal.pone.0139198

**Published:** 2015-10-07

**Authors:** Christopher Zou, Judith P. Andersen

**Affiliations:** Department of Psychology, University of Toronto Mississauga, Mississauga, Canada; The University of Queensland, AUSTRALIA

## Abstract

Few studies have examined the rates of childhood victimization among individuals who identify as “mostly heterosexual” (MH) in comparison to other sexual orientation groups. For the present study, we utilized a more comprehensive assessment of adverse childhood experiences to extend prior literature by examining if MH individuals’ experience of victimization more closely mirrors that of sexual minority individuals or heterosexuals. Heterosexual (n = 422) and LGB (n = 561) and MH (n = 120) participants were recruited online. Respondents completed surveys about their adverse childhood experiences, both maltreatment by adults (e.g., childhood physical, emotional, and sexual abuse and childhood household dysfunction) and peer victimization (i.e., verbal and physical bullying). Specifically, MH individuals were 1.47 times more likely than heterosexuals to report childhood victimization experiences perpetrated by adults. These elevated rates were similar to LGB individuals. Results suggest that rates of victimization of MH groups are more similar to the rates found among LGBs, and are significantly higher than heterosexual groups. Our results support prior research that indicates that an MH identity falls within the umbrella of a sexual minority, yet little is known about unique challenges that this group may face in comparison to other sexual minority groups.

## Introduction

A growing body of evidence indicates that disparities exist between sexual minority individuals and their heterosexual counterparts. One widespread finding is that sexual minority groups consistently show higher prevalence rates of childhood victimization (e.g., physical or sexual abuse, parental neglect, witnessing domestic abuse, all before the age of 18 than their heterosexual peers (e.g., [1–4]). For example, based on a nationally representative sample, Andersen and Blosnich [1] provided evidence that lesbian, gay, and bisexual groups (LGBs) are 60% more likely to have experienced some form of childhood victimization than heterosexuals. Additionally, researchers have also shown that LGBTs report higher rates of peer victimization (i.e., bullying) than their heterosexual peers (e.g., [5–6]). This is a pressing concern for not only researchers, but also the public, as childhood victimization and peer victimization is found to have long-term negative consequences for mental and physical health (e.g., [7–11]).

However, much of the research on disparities in childhood victimization among sexual minorities has focused primarily on gay, lesbian, and bisexual individuals. Few studies have examined the unique challenges that individuals who identify as “mostly heterosexual” (MH), which is sometimes referred to as heteroflexbility [12], may face in comparison with heterosexuals and LGBs (see [5] for a detailed review). MH has recently been established as a distinct orientation group from gay, lesbian, bisexual, and heterosexuals [13–16]. While much of the research on sexual minorities has focused on LGBs, MH individuals comprise a larger proportion of the population than do other sexual minority groups. According to one recent review, up to 7% of individuals identify as MH, which heavily outnumbers the percentage of LGBs [14]. Therefore, it is important for research to examine the unique characteristics and challenges this group may face.

Despite the MH group making up the largest proportion of sexual minorities, many available studies examined the rates of victimization among MHs as a supplementary finding rather than a primary finding [5,17–22]. One study by Austin and colleagues [23], who focused primarily on MHs, compared the rates of victimization between MHs and heterosexuals, but did not include LGBs in their study, so it is unclear how the rates of MHs compare to other sexual minority groups. Additionally, their study included only women, so it is unclear whether their findings replicate in a sample with both genders. In the same vein, Corliss and colleagues [24] examined the rates of familial mental health among MH women and heterosexual women, lacking a gender comparison group.

Among the handful of studies that have examined the rates of childhood victimization among MHs as a secondary topic, most recruited just one gender in their study [17–19]. A greater limitation of past studies is that they often examined just a handful of potential childhood victimization experiences in isolation (e.g., sexual or physical abuse) rather than a comprehensive assessment of a variety of potential adverse childhood experiences that individuals face that may collectively impact their health and well-being over time [25,26]. For the present study, we extend prior research examining childhood victimization disparities among MH individuals and other sexual orientation categories by using a comprehensive assessment of childhood victimization experiences. The objective of this paper is to examine if MH individuals’ experience of victimization more closely mirrors that of sexual minority individuals or heterosexuals using the adverse childhood experiences (ACE) scale [25].

It is useful to examine a variety of childhood victimization experiences in one study to control for the unique characteristics of each specific study (e.g., sample selection, method of assessment, cohort differences). It is difficult to directly compare prevalence rates across studies due to the numerous potential confounds across the different studies. For instance, the prevalence rate of sexual abuse among MHs from one study may differ from the prevalence rate of physical abuse among MHs from another study simply due to the differences in the way sexual orientation was assessed, or when the study was conducted, or where the samples were recruited. A meta-analysis is useful in reducing the differences in external variables of the study by averaging the effects across studies, but the number of studies that have examined the childhood victimization rates of MHs is simply too small to obtain accurate estimates of the prevalence rates of each specific event. While the meta-analysis by Vrangalova and Savin-Williams [27] presented convincing evidence to suggest that MHs experience greater rates of victimization experiences compared with heterosexuals, their analysis does not reveal whether MHs are more likely to experience one type of victimization experience (e.g., physical abuse from parents) than another type of victimization experience (e.g., physical bullying from peers). Additionally, their analysis did not separate childhood victimization from adulthood victimization, which has been shown to have different consequences for long-term health and well-being [7]. In particular, childhood victimization experiences may confer more severe consequences for a child’s health and well-being outcomes than adulthood victimization experiences because they occur at a vulnerable period during the child’s brain development, and the stress response system is particularly sensitive to chaotic family environments, abuse and neglect and peer rejection/harassment [28].

Another limitation of Vrangalova and Savin-William’s [27] meta-analysis is that they solely examined the prevalence rates of victimization experiences between MHs and heterosexuals, and MHs and bisexuals, to establish MHs as a separate category from bisexuals and heterosexuals. While their justification for excluding gays and lesbians is warranted, it remains unclear how the prevalence rates of childhood victimization experiences differ between MHs and gays and lesbians. Vrangolva and Savin-William’s [27] meta-analysis revealed that MHs generally tend to experience less victimization than bisexuals, but how the rates compare to gays and lesbians remains unknown.

### The Present Study

Our aim is to examine the disparities in rates of childhood and peer victimization between LGBs, heterosexuals, and MH individuals. Although bisexuality is perceived to be easier to conceal than homosexuality [29], studies have shown that the rates of childhood victimization experiences among bisexuals equal and sometimes surpass rates of victimization of lesbian and gay individuals [1,7,11,30–32]. One explanation for their elevated risk is that they often face discrimination not only from their heterosexual peers but also their gay/lesbian peers [32,33].

While bisexuals and MHs both share the problem of “double discrimination”, MHs tend to behave more closely to heterosexuals than bisexuals when it comes to sex and relationships. For instance, one study found that a larger proportion of mostly heterosexual boys (6%) and girls (9%) have had a same-sex partner than heterosexual boys (1.5%) and girls (2%). However, this prevalence rate is still much lower than the proportion of bisexual boys (%) and girls (38%) who have had a same-sex partner [21]. This could suggest that MHs should experience fewer victimization experiences because their sexual minority status is less visible. However, as discussed previously, MHs typically show elevated risk of victimization than heterosexuals (e.g., [16]). One explanation could be that environmental and genetic factors that are related to non-heterosexuality are also linked to personality characteristics that may exacerbate one’s risk for experiencing ACE. For instance, the genes that are linked to non-heterosexuality have also been linked to gender nonconformity [16,34], and gender nonconformity has been shown to lead to elevated risks of victimization [35,36].

Therefore, we hypothesize that MH individuals will report equal or higher rates of childhood and peer victimization than gays and lesbians because the MH sexual category is similar to bisexuals in that they both experience double discrimination from both the heterosexual and gay/lesbian groups. In line with prior research we predict that the rates of childhood victimization among MH individuals will be greater than that experienced by heterosexuals. One unique aspect of our study is that we used a comprehensive measure of childhood victimization experiences by utilizing an improved version of the adverse childhood experiences (ACE) questionnaire [25].

## Materials and Methods

### Ethics Statement

This study was reviewed and approved by the University of Toronto Research Ethics Board.

### Sample

Using Amazon’s Mechanical Turk (MTurk; www.mturk.com), we recruited 1,311 participants. MTurk is a tool that is widely used in social science research as a crowdsourcing tool that consists of over 100,000 workers that complete tasks for a small monetary compensation [37]. MTurk workers consist of people from all over the world, but primarily from the United States and India [38]. Works on MTurk are generally slightly younger, more educated, and have slightly lower income levels than the general population from the United States [39]. For the present study, we only allowed participants located in the United States to participate because the climate of LGBT rights vastly differ across the world [40]. Hence, our survey was only visible to MTurk workers who disclosed their location to be in the United States. Results obtained from MTurk workers have been shown to replicate previously established findings in social psychology and physical health [11,41]. In order to ensure that participants paid attention throughout the questionnaire, we embedded three attention checks into the questionnaire, with each attention check appearing evenly throughout the questionnaire. One example attention check asked participants to select “neutral” for a particular question, and any participants who did not answer “neutral” would have failed the attention check. To retain as much statistical power as possible, we included participants who passed at least two attention checks in the data analysis. The pattern of results remained the same even when retaining only the participants who passed all three attention checks. After filtering the data for participants who did not meet the requirement, we retained 1,145 (87%) of the participants. For the gender variable, 636 identified as female, 484 as male, 7 as transgender, 1 as transwoman, 3 as transman, 2 as other identified, and 10 identified as the “other” category. In an unfortunate oversight, wee did not obtain sexual orientation information from individuals who did not identify as male or female, necessitating their exclusion from data analysis. 422 participants identified as exclusively heterosexual, 120 mostly heterosexual, 323 bisexual, 135 gay, 103 lesbian, and 16 identified as questioning. Due to the smaller sample size, we omitted participants who identified as questioning. By filtering by gender and sexual identity, this left an analytic sample of 1,103 participants.

### Data Collection

We posted two separate postings on MTurk with one post targeting anyone who was willing to participate and another post that targeted sexual minorities. Participants filled out a series of questionnaires about their childhood experiences, which included measures of peer and childhood victimization. The questionnaire took approximately one hour to complete. Once the participants completed the questionnaire, they were debriefed and compensated with two dollars for participating in the study. Although this may seem low, this amount is comparable to what MTurk workers are paid for both research and non-research work of similar length [42]. We found it most reasonable to stay within the ‘going rate’ of pay on MTurk. Additionally, compensation rates on MTurk have been shown to not influence the outcomes of the study, as compared to similar, non-MTurk studies paid at a higher rate [37]. This study was approved by the institutional ethics board at the University of Toronto.

### Measures

#### Adverse childhood events

Participant’s experiences of childhood victimization were assessed by asking them to indicate if they had experienced any of fourteen adverse childhood events using the Adverse Childhood Events (ACE) scale [25]. The ACE scale was developed by Felitti and colleagues (1998) in collaboration with the Chronic Disease Prevention and Health Promotion (CDC) to assess people’s experiences of childhood victimization. The ACE scale assesses factors beyond sexual and physical abuse such as familial substance abuse, parental incarceration, and family mental illness. These additional risk factors have traditionally not been assessed using scales other than the ACE. Dube and colleagues [43] conducted a test-retest reliability of the ACE questionnaire in an examination 658 participants over two time periods. The authors report Kappa coefficients for each question separately, with a range between .52 and .72 [43]. As established in the literature, Kappa values between .40 and .75 represent good agreement [44]. However, the original ACE scale omits domains that have been shown to be important for long-term well-being and health [26]. One important domain is peer victimization (i.e., bullying), which has been shown to be highly prevalent in schools (29.0% in the United States [45]). We included this domain by adding two additional items (verbal bullying, physical bullying) to improve on the original ACE scale. Each ACE event reported was summed to compute an overall ACE score from 0 to 16.

#### Gender

Gender was assessed with a one-item measure that asked participants to indicate their gender as male, female, transgender, transwoman, transman, other identified, or other, “please define”.

#### Sexual identity

Sexual identity was assessed with a one-item measure that asked participants to indicate if they identify as exclusively heterosexual, mostly heterosexual, bisexual, gay, lesbian, or questioning. Our group of interest for the present study is mostly heterosexuals, so this group was coded as the reference group to which other groups were compared.

#### Demographic variables

Participants were also asked to report their age, and their race (i.e., white, Asian, black, Latino, other). For the race variable, white was coded as the reference group because this was the largest racial group in our sample.

### Data Analysis

Gender differences have been consistently found in victimization experiences (e.g., [46]). Thus, comparisons were only made between the same gender groups unless stated otherwise. One-way ANOVAs were used to compare mean differences between the groups. Post-hoc t-test comparisons were made using a Bonferonni correction for multiple comparisons. Independent Samples Kruskal-Wallis tests were used to examine differences in frequencies between the groups. Subsequent Kruskal-Wallis tests were conducted to make post-hoc pairwise comparisons with Bonferonni adjustments to take multiple comparisons into account. To avoid confounding gender with sexual identity, we merged the gay and lesbian groups together and grouped both genders of MHs, heterosexuals, and bisexuals together for the regression analysis. To account for ACE as a count variable, we conducted a Poisson regression to examine the association between sexual identity and ACE while controlling for age (i.e. cohort effects) and gender. All the analyses were conducted on SPSS Version 22.

## Results

### Sample Characteristics

The average age of the sample was 32.54 (SD = 12.0) years, which ranged from 18 to 75 years of age. There were significant differences in age among the female groups (F (3, 624) = 40.96, *p* < .001). In particular, the female heterosexual group was significantly older than the female MH, bisexual, and lesbian groups (*p* < .001 for all comparisons). Additionally, female bisexuals were significantly younger than the lesbians (*p* = .019). There were also significant differences in the distribution of race across the four groups (chi-square (3) = 7.97, *p* = .047). However, no specific pairwise comparisons were significant.

There were also significant differences in age among the male groups (F (3, 470) = 5.52, *p* = .001). Specifically, the male heterosexual group was older than the MHs and the bisexuals (*p* = .03 and *p* = .003, respectively). The education level significantly differed across the sexual orientation groups for men (chi-square (3) = 10.79, *p* = .013). Specifically, heterosexual men were generally more educated than mostly heterosexual men (*p* = .008). Table 1 provides detailed demographic characteristics across all four sexual identity groups. Our age and education distributions were comparable to those found in prior studies conducted on MTurk [11,39].

**Table 1:** Demographic Characteristics of Gay/Lesbian, Bisexual, and Mostly Heterosexual Groups.

	Heterosexual (n = 422)		Gay/Lesbian (n = 238)		Bisexual (n = 323)		Mostly Heterosexual (n = 120)	
	M (n = 189)	F (n = 233)	G (n = 135)	L (n = 103)	M (n = 111)	F (n = 212)	M (n = 39)	F (n = 81)
Age (SD) (M**/F***)	34.41 (12.75)	39.09 (14.34)	31.95 (11.81)	31.84 (10.48)	29.48 (10.11)	27.87 (7.99)	28.64 (11.67)	29.54 (8.29)
Race (%) (F*)								
Caucasian/White	154 (81.5)	180 (77.9)	110 (81.5)	69 (67.6)	90 (81.1)	151 (71.6)	33 (84.6)	65 (81.3)
Black/African American	9 (4.8)	24 (10.4)	5 (3.7)	14 (13.7)	2 (1.8)	19 (9.0)	2 (5.1)	2 (2.5)
Asian/Pacific Islander	15 (7.9)	12 (5.2)	6 (4.4)	5 (4.9)	8 (7.2)	6 (2.8)	2 (5.1)	6 (7.5)
Latino/Hispanic	7 (3.7)	4 (1.7)	6 (4.4)	9 (8.8)	7 (6.3)	17 (8.1)	2 (5.1)	1 (1.3)
Other	4 (2.1)	11 (4.8)	8 (5.9)	5 (4.9)	4 (3.6)	18 (8.5)	0 (0.0)	6 (7.5)
Education (%) (M*)								
Highschool/GED equivalent	34 (18.0)	28 (12.0)	12 (9.1)	19 (18.4)	18 (16.4)	39 (18.5)	10 (25.6)	18 (22.2)
Some post-secondary	55 (29.1)	83 (35.6)	45 (34.1)	31 (30.1)	35 (31.8)	74 (35.1)	16 (41.0)	29 (35.8)
Completed college/university	77 (40.7)	97 (41.6)	50 (37.9)	38 (36.9)	44 (40.0)	74 (35.1)	11 (28.2)	29 (35.8)
Master’s degree/Doctoral	23 (12.2)	25 (10.7)	25 (18.9)	15 (14.6)	13 (11.8)	24 (11.3)	2 (5.1)	5 (6.1)

Note: M = male, F = female, significance corresponds to significant differences between the groups at *p < .05, ** *p* < .01, *** *p* < .001

### Differences in Victimization Experiences

Overall ACE scores significantly differed across sexual orientations for men (F(3, 470) = 10.74, *p* < .001). Specifically, heterosexual men reported lower ACE scores than bisexuals and gays (*p* < .001 for both comparisons). Similarly, overall ACE scores significantly differed across sexual orientations for women (F(3, 625) = 9.60, *p* < .001). Specifically, heterosexual women reported fewer ACE than mostly heterosexual women and bisexual women (*p* = .005 and *p* < .001, respectively). Table 2 displays the prevalence rates of victimization experiences across the sexual identity groups.

**Table 2:** Prevalence Rates of Victimization among Gay/Lesbian, Bisexual, Heterosexual, and Mostly Heterosexual Groups.

	Heterosexual (n = 422)		Gay/Lesbian (n = 238)		Bisexual (n = 323)		Mostly Heterosexual (n = 120)	
Item of ACE	M (n = 189)	F (n = 233)	G (n = 135)	L (n = 103)	M (n = 111)	F (n = 212)	M (n = 39)	F (n = 81)
**Verbal or Physical Abuse (F** *** **/M** ** **)**								
1. Did a parent or other adult in the household often or very often swear at you, insult you, put you down, or humiliate you? Or act in a way that made you afraid that you might be physical hurt?	37 (19.6)	62 (26.6)	51 (37.8)	31 (30.1)	33 (29.7)	86 (40.6)	9 (23.1)	35 (43.2)
2. Did a parent or other adult in the household often or very often push, grab, slap or throw something at you?	19 (10.1)	33 (14.2)	27 (10.1)	22 (21.4)	24 (21.6)	58 (27.4)	4 (10.3)	22 (27.2)
3. Did a parent or other adult in the household often or very often ever hit you so hard that you had marks or were injured?	11 (5.8)	28 (12.0)	18 (13.3)	16 (15.5)	15 (13.5)	37 (17.5)	2 (5.1)	14 (17.3)
**Sexual Abuse (F** *** **/M** *** **)**								
4. Did an adult or person at least 5 years older than you, before you were age 16, ever touch, or fondle you or have you touch their body in a sexual way?	8 (4.2)	32 (13.7)	26 (19.3)	19 (18.4)	10 (9.0)	64 (30.2)	2 (5.1)	16 (19.8)
5. Did an adult or person at least 5 years older than you, before you were age 16, ever attempt or actually have oral, anal, or vaginal intercourse with you?	5 (2.6)	21 (9.0)	14 (10.4)	13 (12.6)	8 (7.2)	36 (17.0)	1 (2.6)	9 (11.1)
**Physical or Emotional Neglect (F** * **/M** *** **)**								
6. Did you often or very often feel that no one in your family loved you or thought you were important or special?	25 (13.2)	68 (29.2)	37 (27.4)	33 (32.0)	36 (32.4)	87 (41.0)	7 (17.9)	26 (32.1)
7. Did you often or very often feel that your family didn’t look out for each other, feel close to each other, or support each other?	32 (16.9)	69 (29.6)	35 (25.9)	33 (32.0)	35 (31.5)	83 (39.2)	9 (23.1)	30 (37.0)
8. Did you often or very often feel that you didn’t have enough to eat, had to wear dirty clothes, and had no one to protect you?	14 (7.4)	23 (9.9)	10 (7.4)	14 (13.6)	17 (15.3)	31 (14.6)	3 (7.7)	14 (17.3)
9. Did you often or very often feel that your parents were too drunk or high to take care of you or take you to the doctor if you needed it?	5 (2.6)	13 (5.6)	15 (11.1)	7 (6.8)	9 (8.1)	22 (10.4)	4 (10.3)	11 (13.6)
**Household Dysfunction (F** *** **/M** *** **)**								
10. Were your parents ever separated or divorced?	59 (31.2)	72 (30.9)	46 (34.1)	35 (34.0)	41 (36.9)	102 (48.1)	16 (41.0)	40 (49.4)
11. Was your mother or stepmother often or very often pushed, grabbed, slapped, or had something thrown at her?	7 (3.7)	24 (10.3)	23 (17.0)	15 (14.6)	15 (13.5)	28 (13.2)	2 (5.1)	13 (16.0)
12. Did you live with anyone who was a problem drink or alcoholic or who used street drugs?	33 (17.5)	60 (25.8)	51 (37.8)	28 (27.2)	33 (29.7)	80 (37.7)	12 (30.8)	23 (28.4)
13. Was a household member depressed or mentally ill, or did a household member attempt suicide?	23 (12.2)	59 (25.3)	36 (26.7)	31 (30.1)	39 (35.1)	76 (35.8)	9 (23.1)	39 (48.1)
14. Did a household member go to prison?	5 (2.6)	9 (3.9)	10 (7.4)	7 (6.8)	9 (8.1)	15 (7.1)	3 (7.7)	8 (9.9)
**School Bullying (F** *** **/M** ** **)**								
15. Were you ever verbally bullied or harassed (called names, teased, or threatened) during high school?	84 (44.9)	89 (38.4)	89 (66.4)	57 (55.9)	60 (54.1)	125 (59.5)	23 (59.0)	46 (56.8)
16. Were you ever physically bullied or harassed (pushed, tripped, had objects thrown at you, punched etc.) during high school?	46 (24.7)	31 (13.4)	50 (37.0)	23 (22.5)	34 (30.6)	59 (27.8)	11 (28.2)	19 (23.8)
Overall ACE (M, SD)	2.18 (2.48)	2.97 (3.36)	3.98 (3.48)	3.73 (3.53)	3.77 (3.73)	4.67 (3.61)	3.00 (2.64)	4.51 (3.68)

Note: M = male, F = female, significance corresponds to chi-square tests of prevalence rates

*p < .05,

** *p* < .01,

*** *p* < .001

In order to examine potential differences across sexual orientations for specific types of victimization experiences, we categorized the 16 items of the ACE scale into 4 groups: verbal or physical abuse (items 1, 2, 3), sexual abuse (items 4, 5), physical or emotional neglect (items 6, 7, 8, 9), household dysfunction (items 10, 11, 12, 13, 14), and school bullying (items 15, 16). Each comparison was conducted by each gender to control for any gender differences in prevalence rates of childhood victimization experiences.

The prevalence rates of verbal or physical abuse among females differed across sexual orientations (chi-square (3) = 16.53, *p* = .001). Specifically, heterosexual women were less likely to report child verbal or physical abuse from a parent than mostly heterosexual women and bisexual women (*p* = .028 and *p* = .002, respectively). The prevalence rates of child sexual abuse also differed (chi-square (3) = 18.10, *p* < .001), whereby heterosexual women reported fewer child sexual abuse experiences than bisexual women (*p* < .001). The prevalence rates of neglect also differed among women across the sexual orientations groups (chi-square (3) = 10.49, *p* = .015), where once again, heterosexual women reported fewer neglect experiences than bisexual women (*p* = .010). Rates of household dysfunction also differed across the sexual orientation groups among women (chi-square (3) = 21.30, *p* < .001). Specifically, heterosexual women reported fewer household dysfunction experiences than bisexual women and mostly heterosexual women (*p* < .001 and *p* = .004, respectively). The prevalence rates of school bullying also differed across sexual orientation among women (chi-square (3) = 26.10, *p* < .001), whereby heterosexual women reported fewer instances of bullying than lesbians, bisexual women, and mostly heterosexual women (*p* = .021, *p* < .001, *p* = .016). We did not find significant differences between any of the sexual minority groups for women.

The prevalence rates of child physical abuse significantly differed across sexual orientations for men (chi-square (3) = 13.85, *p* = .003). Specifically, heterosexual men reported fewer cases of physical abuse than gay men (*p* = .003). The prevalence rates of child sexual abuse also significantly differed across sexual orientations (chi-square (3) = 22.57, *p* < .001). Heterosexual men were less likely to report child sexual abuse than gay men (*p* < .001). Additionally, gay men were more likely to report child sexual abuse than mostly heterosexual men and bisexual men (*p* = .041 and *p* = .034, respectively). Rates of neglect also differed across sexual orientation among men (chi-square (3) = 19.86, *p* < .001). Heterosexual men reported fewer instances of neglect than gay men and bisexual men (*p* = .019 and *p* < .001, respectively). Rates of household dysfunction also differed across sexual orientation among men (chi-square (3) = 16.75, *p* = .001). Specifically, heterosexual men reported fewer household dysfunction events than bisexual men and gay men (*p* = .046 and *p* = .001, respectively). The prevalence rates of school bullying also significantly differed across sexual orientation among men (chi-square (3) = 13.40, *p* = .004). Specifically, heterosexual men were less likely to report being bullied in school than gay men (*p* = .002).

We also conducted an analysis comparing the regression rates across sexual orientations in a regression model to control for gender, age (cohort), and race because these variables have been shown to influence the experiences and prevalence rates of childhood victimization experiences [47–49]. ACE is a count variable, so we conducted a negative binomial regression model to see if sexual identity predicted ACE scores. Sexual identity was a significant predictor of ACE scores (Wald chi-square (3) = 35.99, p < .001). Specifically, mostly heterosexuals were 1.47 times (95% CI = (1.16, 1.87)) more likely to report more ACE events than heterosexuals. Table 3 provides a summary of the negative binomial regression model.

**Table 3:** Regression Models Predicting ACE from Sexual Identity.

Variable	ACE^1^	*p*-value
Age	1.00 [1.00, 1.01]	.339
Gender^2^	.68 [.54, .86]	.001
Race (Asian)	.55 [.40, .76]	<.001
Race (Black)	.85 [.64, 1.11]	.230
Race (Latino)	1.04 [.76, 1.43]	.802
Race (Other)	1.47 [1.08, 1.99]	.059
Sexual Identity (Gay/Lesbian)	1.04 [.81, 1.34]	.750
Sexual Identity (Bisexual)	1.11 [.88, 1.41]	.382
Sexual Identity (Heterosexual)	.68 [.54, .86]	.001

^1^ Negative binomial regression was used

^2^ 0 = female, 1 = male

Note: All races were compared to White/Caucasian. All sexual identities were compared to MHs.

## Discussion

While there is widespread evidence to demonstrate that LGBs experience higher rates of childhood and peer victimization than heterosexuals, it was unclear from the literature whether rates of victimization among MH individuals will be comparable to that of heterosexuals, or of LGBs. Based on the present study, the data suggests that rates of victimization of MH groups are more similar to the rates found among LGBs, and are significantly higher than heterosexual groups. When examining each gender separately, mostly heterosexual women reported more adverse childhood events than heterosexual women, but their rates did not differ from those of bisexual women and lesbians. On the other hand, we did not find any significant difference in the prevalence rates of mostly heterosexual men and any of the other sexual orientation groups. This suggests that mostly heterosexual women may be particularly vulnerable to experiencing victimization in childhood or are more open to reporting victimization experiences.

Our study extended the findings from a handful of previous studies that have examined the victimization rates of MH. First, our study focused directly on childhood victimization experiences, which have been shown to have particularly detrimental consequences for long-term health and well-being [7]. Second, our study examined a wide range of childhood victimization experiences in a single study using the improved ACE scale including peer bullying, which allows for direct comparisons between difference childhood victimization events. Including peer bullying highlights a broader range of victimization experiences that sexual minorities and MH experience. This study suggests that the rates of child physical/verbal abuse, household dysfunction, and peer bullying significantly differed between heterosexual and mostly heterosexual women. Further replication is necessary to establish these differences across sexual orientation groups.

Another advantage of our study over previous studies is that we examined sexual orientation across genders. This allowed us to examine differences in prevalence rates that are attributed to sexual orientation rather than gender. Additionally, by analyzing the differences in sexual orientation across genders, we were also able to examine differences between genders while controlling for sexual orientation. For example, mostly heterosexual women reported more victimization experiences than mostly heterosexual men for 16 out of 16 comparisons on each of the ACE items. This suggests that mostly heterosexual women are more at risk of experiencing childhood victimization than mostly heterosexual men or more open to reporting it. This gender by sexual orientation analysis would not be possible if our study did not recruit both genders, and did not separate our sample by gender and sexual orientation.

Examining causal reasons for MH experiencing higher rates of victimization are beyond the scope of this study. However, evidence from studies of the treatment of non-conforming individuals may shed some insight into why MH individuals experience prevalence rates of victimization similar to LGB groups. Early childhood and late adolescence is a time when gender roles and social behaviors are very salient for children and teens [50]. Individuals who counter these strict gender and social norms are often severely ‘policed’ or sanctioned by parents and peers [51,52]. For example, a male who wears makeup and identifies with a ‘counter-society’ movement (e.g., punk, goth) may be targeted for bullying or victimization due to non-conforming behaviors or attitudes, irrespective of sexual orientation [53]. Non-conforming individuals may be less likely to conform to the strict norms of heterosexuality, and thus more willing to identify as MH, even if they have not had a same sex sexual relationship. Some individuals may wonder why an MH person would be targeted form abuse, particularly as it may be easier to ‘pass’ as a heterosexual individual. In order to tease apart causes of victimization among MH compared to LGB, it would be important to conduct a study examining the specific reasons for victimization experiences (i.e., sexual orientation, gender non-conforming, or general societal non conforming behaviors and attitudes). These questions are an important avenue for future research.

Although MH individuals do comprise the largest group of sexual minority individuals in general, in the present study, MH individuals comprised of the smallest sexual minority group when compared with LGBs. This may be related to our assessment of whether an individual was MH, because the method of assessment can heavily influence rates of MHs [14]. In our study, we categorized an individual as MH if they identified as MH out of a number of different categories of sexual identity (i.e., gay, lesbian, questioning, exclusively heterosexual). Individuals who identify as “mostly heterosexuals” are typically committed and certain in their MH identity [14,15].

It is also possible that the rates of victimization among MHs may have differed if we used a different sexual orientation indicator (e.g., arousal, desire, behavior). In Vrangalova and Savin-William’s [27] meta-analysis of MHs and rates of victimization, MHs had lower rates of victimization than bisexuals, which is contrary to our findings that showed no difference in rates of victimization between MHs and bisexuals. However, Vrangalova and Savin-William [27] combined all the studies that used different sexual orientation indicators. As they acknowledged in their paper, it is possible that the level of risk may differ depending on which indicator is used to assess sexual orientation. For instance, individuals who identify with the MH status are likely aware that they do not fit in with the heterosexual majority, and this awareness may lead them to feel and act isolated, which can increase their likelihood of being victimized [54]. However, if the MH category was based on a slight desire for same-sex partners, then it is possible that some MHs may not necessarily see their own desires as being different from the heterosexual norm and may feel as though they fit in with the heterosexual group. MH individuals with an absence of the awareness that they are different from their peers, may be less likely targets of victimization. Future studies should examine how the different indicators of sexual orientation influence rates of victimization.

Additionally, MH is relatively an unknown sexual categorization among the public, and it has only recently been established as a distinct category in research. Therefore, it is likely that many MH individuals categorized themselves as being bisexual or heterosexual, because these categories are better understood. In future studies, it would be beneficial to explicitly report the high prevalence of MHs to participants, so that individuals who fall in this category will be more likely to identify with this group. Another potential method to assess sexual identity is to allow individuals to identify their sexual orientation on a continuum, such as on a Kinsey Scale [14,55]. Continuum scales allow researchers to appropriately categorize individuals based on their conceptualization of MH status. However, such a scale may not necessarily capture all the possible sexual identity categories, such as individuals who are “questioning” [56], “pansexual/polysexual” [57], and “asexual” [58], which are orientations that are difficult to assess on a continuum like the Kinsey Scale [59]. Despite the limitations of our assessment of sexual identity, we were able to gather a large enough sample of MHs (>100) that allowed for the detection of medium effect-sizes with enough power to detect effects [60].

### Limitations

There are limitations of this study that open avenues for future research. First, our focused on the presence or absence of both ACE and peer victimization events. We did not examine the details of each event. Victimization events can vary in age of onset, severity, and frequency, which can increase the range in which differences may be detected between the different sexual identity groups [61]. Studies have found that sexual minorities generally experience more severe and frequent forms of sexual abuse [61]. However, no research has examined if the characteristics of victimization differ between sexual minority groups by gender. This remains an open avenue for future researchers to examine.

Second, our results were based on self-reported experiences of victimization. In order to reduce potential biases in self-reports of victimization experiences due to fear of embarrassment or shame, we conducted this study on an online medium where participants were able to complete the questionnaires in the privacy of their own homes. Additionally, through the use of Mturk, participants can complete the questionnaires without revealing any identifiable information to the experimenters. A more serious concern may be that participants may not accurately recall their victimization experiences due to memory errors. However, studies report that memory for the occurrence of traumatic events remain fairly accurate over a long period of time [62–66], while the accuracy of the details of these events are somewhat controversial [67,68].

Third, due to the limited amount of available space in our survey, our assessment of ACE and bullying do not capture the vast range of victimization experiences that people may have experienced. For example, the ACE scale does not include experiences like low socioeconomic status and poor school performance, which can be added to the ACE scale to improve its predictive validity [69]. Our current measure of bullying also did not capture other forms of bullying such as ostracism [70] and more recent popular form of cyber bullying [71]. We encourage future studies of victimization experiences to include a wider range of items to assess more categories of victimization.

Fourth, while the primary focus of the paper was on mostly heterosexuals, there is some evidence that mostly gay/lesbian should also be a distinct category of its own that is different from exclusively gay/lesbian [14]. However, there is much less work that has examined mostly gay/lesbian groups, and thus is a wide-open avenue for future researchers to explore. Echoing the suggestions by Savin-Williams and Vrangalova [27], we strongly encourage future researchers to adopt at least five categories of sexual orientation (heterosexual, mostly heterosexual, bisexual, mostly gay/lesbian, gay/lesbian) to better capture the sexual orientation categories of the general public.

Lastly, our data was cross-sectional so we were not able to determine the cause of the disparities in early victimization experiences. One possibility is that sexual identity leads to greater rates of childhood victimization due to peers and adults targeting a child who displays gender non-conforming behaviors early on in childhood or adolescence [35, 36]. On the other hand, some researchers have suggested that early experiences of victimization can influence one’s sexual identity [52]. However, this research is controversial given that sexual orientation is most likely determined through the interplay of biology and environmental experiences [72]. Further, if victimization were to cause sexual orientation status there would be a much greater prevalence of sexual minorities, given the rates of childhood abuse and neglect are around 40% among women [73]. While the issue of causality is important in understanding disparities in victimization, the nature of our data does not allow us to test the direction of the relationship between sexual orientation and early victimization experiences.

### Conclusion

While MH individuals make up the largest group of sexual minorities, little research has focused on this group [13]. Our study adds to the literature on sexual minorities by examining the unique characteristics and experiences of MH individuals around disparities in rates of childhood and peer victimization. We found that thee elevated levels of early victimization among MH individuals are similar to that of LGBs. A recent review has demonstrated that health disparities exist between MHs and heterosexuals, where MHs report higher levels of mental and physical health symptoms, and health risk behaviors such as smoking and drinking [24]. Based on the widespread evidence linking early childhood victimization experiences, health risk behaviors and mental and physical health conditions (e.g., [7,74]), it is possible that childhood victimization experiences may explain some of the health disparities observed between MHs and heterosexuals. This is a crucial avenue for future research in order to create effective interventions to reduce these disparities.
